# Shift of Creep Mechanism in Nanocrystalline NiAl Alloy

**DOI:** 10.3390/ma12162508

**Published:** 2019-08-07

**Authors:** Zhihui Sun, Baoshu Liu, Chenwei He, Lu Xie, Qing Peng

**Affiliations:** 1 School of Mechanical Engineering, University of Science and Technology Beijing, Beijing 100083, China; 2 Reactor Engineering and safety research center, China nuclear power technology research institute Co., Shenzhen Ltd., Shenzhen 518031, China; 3 Nuclear Engineering and Radiological Sciences, University of Michigan, Ann Arbor, MI 48108, USA

**Keywords:** creep mechanism, molecular dynamics simulation, nanocrystalline, NiAl

## Abstract

We have examined the effects of temperature, stress, and grain size on the creep process including creep strain, crystal structure, dislocations and diffusions of nanocrystalline NiAl alloy through molecular dynamics simulations. A smaller grain size accelerates the creep process due to the large volume fraction of grain boundaries. Higher temperatures and stress levels also speed up this process in terms of dislocation changes and atom diffusion. In both primary creep and steady-state creep stages, atomic diffusion at the grain boundary could be seen and the dislocation density increased gradually, indicating that the creep mechanism at these stages is Coble creep controlled by grain boundary diffusion while accompanied by dislocation nucleation. When the model enters the tertiary creep stage, it can be observed that the diffusion of atoms in the grain boundary and in the crystal and the dislocation density gradually decreases, implying that the creep mechanisms at this stage are Coble creep, controlled by grain boundary diffusion, and Nabarro–Herring creep, controlled by lattice diffusion.

## 1. Introduction

The intermetallic compound NiAl is of great importance due to its unique characteristics, including its high melting point, low density, outstanding thermal conductivity and excellent oxidation resistance [1,2,3]. It is expected to substitute nickel-based superalloys as the next generation of high-temperature structural materials. Its creep properties are crucial for the high-temperature applications of NiAl; however, these are not fully understood.

Raj et al. [4] have assessed the creep mechanism of fine-grained polycrystalline NiAl under different creep stages and found that the experimental creep rates are several orders of magnitude lower than those predicted by theoretical models. Xiao et al. [5] have adopted mechanical alloying and high-temperature hot pressing processes to synthesize NiAl nanocrystalline materials. Their findings show that the strength and plasticity of nanocrystalline NiAl are higher than those of coarse-grained NiAl. However, the plasticity of NiAl is still lower than that of nickel-based superalloys. According to Pal [6], the plasticity of nanocrystalline metal can be enhanced by further grain refinement near the critical size of 20–25 nm. Therefore, whether the nanocrystalline NiAl fulfills the working requirements by further grain refinement has become a matter of concern. Moreover, the creep process and creep mechanism of nanocrystalline NiAl alloys (grain size ≤10 nm) remains largely unknown.

Due to the challenges in material preparation and experimental conditions, the nature of the physical phenomena at the atomic level is still elusive. Molecular dynamics (MD) simulations have been recognized as a reliable method for mechanism investigations [7], including studying the creep process of nanomaterials and elucidating the creep mechanisms. For instance, through MD simulation, Keblinski et al. [8] suggest that the Coble creep mechanism plays a major role during the creep deformation of nanocrystalline materials (grain size ≤10 nm). Yamakov et al. [9] have investigated grain boundary diffusion in the creep process of nanocrystalline palladium at elevated temperatures. They have reported that grain boundary sliding may serve as an accommodation mechanism for grain boundary diffusion creep. Besides this, Millett et al. [10] have studied the diffusional creep in nanocrystalline molybdenum. Their MD simulation results indicate that the overall deformation is dominated by grain boundary diffusion and lattice diffusion, and vacancies are found to be generated from the grain boundaries into the grain interiors. Additionally, their findings reveal that lattice diffusion represents one of the most important mechanisms underlying creep. Despite the inherent limitation of high strain rates, the underlying creep mechanisms can be identified by MD simulations at a specific timescale. Previous simulation results have shown good agreement with phenomenological models and experimental data [11].

In this study, for the first time, we investigated the effects of temperature, stress and grain size on the creep of nanocrystalline NiAl alloy (grain size ≤10 nm) through MD simulations. We have focused on the creep strain, crystal structure, dislocations and diffusions. The underlying creep mechanisms at different creep stages were explored. Specifically, we study the mechanism of creep properties at the micro–nano scale. For MD simulations, the time scale is relatively short and the system is not large enough, which are the normal limitations of molecular dynamics. Since the geometric scale and time scale of the molecular dynamics method are different from the actual experiment, the stress level and strain rate are much higher than the actual experiment [8,9,12,13,14]. Although creep is a time-dependent phenomenon that usually occurs on time scales beyond the MD range, creep behavior and microstructure evolution can be observed even at high strain rates in MD simulations. In this research, the creep curve of the material shows the same characteristics as the three stages of actual creep—the primary creep phase, steady-state creep phase and tertiary creep phase—and the deformation mechanism obtained by the MD method is consistent with the high-temperature creep mechanism. Previous studies into creep have also proved the rationality of the MD method, and the time scale and stress level used in their research are in the same order of magnitude as this study [6,8,9,12,13,14].

## 2. Simulation Model and Process

**Figure 1:**
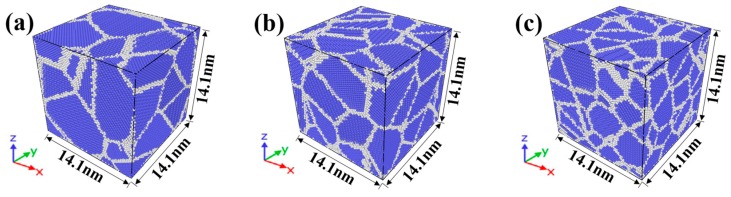
Nanocrystalline NiAl models with different grain sizes of (**a**) 6.5, (**b**) 4.5 and (**c**) 3.8 nm.

Three nanocrystalline NiAl models were established by Atomsk software [15] using a Voronoi construction [16]. Figure 1 shows the three model geometries for MD simulations. The blue part is the Body Centered Cubic (BCC) crystal of NiAl, and the gray part is the grain boundary structure. Nanocrystalline NiAl models contained 10, 30 and 50 grains in a body-centered cubic of random orientation, and their grain sizes were 6.5, 4.5, 3.8 nm, respectively. There are 234,467, 234,471 and 234,300 atoms in each model, respectively. All the three models demonstrated the same dimensions (14.1 nm × 14.1 nm × 14.1 nm).

Periodic boundary conditions were used in *x*, *y* and *z* directions. The embedded atom method (EAM) potential was used for NiAl, as described previously, and the potential proposed was constructed using experimental data and a large set of ab initio structural energies [17]. The time step was set to be 1 fs. Creep simulation was performed using an isothermal–isobaric (NPT) ensemble with zero pressure in both *x* and *y* directions, and a constant tensile stress was applied to the *z* direction controlled by the Parrinello–Rahman method [18]. The simulation time of nanocrystalline NiAl models was 200 ps using the same relaxation and loading method. All simulations were carried out at high temperature. The Large-Scale Atomic/Molecular Massively Parallel Simulator (LAMMPS) MD code [19] and Ovito visualization tool [20] were used for atomistic simulations. The atomic configurations and their evolution were analyzed by a centro-symmetry parameter (CSP) in order to provide the details of microstructure evolution during creep simulation in nanocrystalline NiAl models [21]. The radial distribution function was used to reflect the degrees of amorphization in the three models. The dislocation extraction algorithm (DXA) was used to reveal the dislocation density of the three models [22]. The number of vacancies was calculated at different time steps of the creep deformation process, simulated by Wigner–Seitz defect analysis [23]. The mean square displacement (MSD) was calculated to obtain the values regarding atomic diffusion [24].

## 3. Results and Discussion

### 3.1. Creep Phenomenon and Possible Reasons

**Figure 2:**
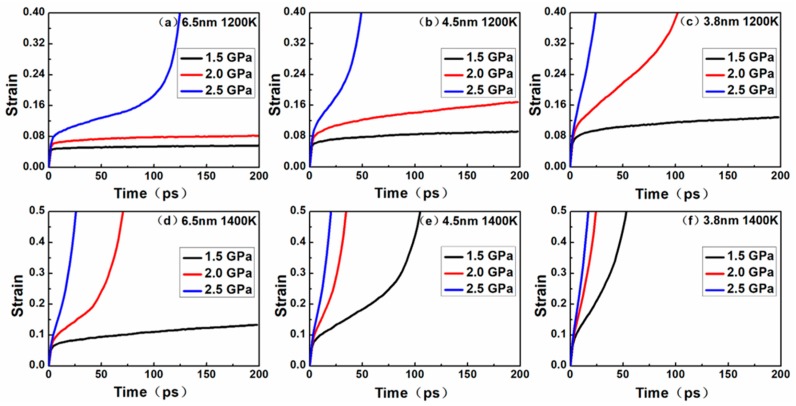
Time versus creep strain curves for the nanocrystalline models with grain sizes of (**a**) 6.5 nm at 1200 K; (**b**) 4.5 nm at 1200 K; (**c**) 3.8 nm at 1200 K; (**d**) 6.5 nm at 1400 K; (**e**) 4.5 nm at 1400 K; (**f**) 3.8 nm at 1400 K under the stress levels of 1.5, 2.0 and 2.5 GPa.

To systematically investigate the effects of temperature, stress and grain size on the creep process, three different levels of stress (1.5, 2.0 and 2.5 GPa) were applied on the three different grain size models (6.5, 4.5 and 3.8 nm) at different temperatures (1200 and 1400 K). Figure 2 shows the evolution of strain with time for nanocrystalline NiAl.

In the creep curves of Figure 2, the slope of the curves represented the strain rate during creep. The strain rate decreased with time, representing the primary creep phase, characterized by the slope of the curve tending to be flat. The strain rate remained constant with time, representing the steady-state creep phase, characterized by the slope of the curve being constant. The strain rate increased with time, representing the tertiary creep phase, characterized by a steep increase in the slope of the curve.

All the curves exhibited a short elastic regime for the first few picoseconds, followed by entering the primary creep and steady-state (secondary) creep stages, and most curves entered the tertiary creep stage. At a temperature of 1200 K, the creep phenomenon of different grains under different stresses was as follows: for the nanocrystalline NiAl model with a grain size of 6.5 nm, no tertiary creep was observed under 1.5 Gpa and 2.0 GPa, except for that under 2.5 GPa. For the nanocrystalline NiAl model with a grain size of 4.5 nm, the phenomenon is consistent with the grain size of 6.5 nm, while the strain rate is higher. For the nanocrystalline NiAl model with a grain size of 3.8 nm, no tertiary creep was observed under 1.5 Gpa, except for that under 2.0 GPa and 2.5 GPa. When the temperature was raised to 1400 K, the creep phenomenon of different grains under different stresses was as follows: for the nanocrystalline NiAl model with a grain size of 6.5 nm, no tertiary creep was observed under 1.5 Gpa, except for that under 2.0 GPa and 2.5 GPa. For the nanocrystalline NiAl model with a grain size of 4.5 nm and 3.8 nm, tertiary creeps were observed under all stress levels, and the smaller the grain size, the faster the strain rate.

It was obvious that higher temperatures and stress levels might accelerate the occurrence of tertiary creep, as well as increase the rate of creep process. Besides, the same phenomenon was observed when the grain size decreased. These observations may be explained by the following two aspects.

On the one hand, under higher temperatures and stress levels, nanocrystalline NiAl can have several types of defects such as vacancies, dislocations and grain boundaries, leading to an obvious creep process. As temperature increases, the frequencies and amplitudes of atomic vibrations become higher. The increased temperature allows for the atoms to pass over the energy barrier. Hence, the diffusion and dislocation activity may be accelerated during creep process. As stress increases, the equilibrium concentration of vacancies is altered in nanocrystalline NiAl. If grain boundaries and dislocations are applied as tensile stress, the equilibrium concentration of vacancies may be increased [25]. Thus, if a vacancy concentration gradient occurs in a crystal, the vacancies can diffuse along rapid diffusion paths, such as grain boundaries or dislocations. At the same time, the diffusion rate may be increased because atoms are more likely to overcome the energy barrier under high stress levels. Additionally, higher stress levels enhance the nucleation of dislocations and their glide velocity.

On the other hand, the decreases in the grain size of a model increase the volume fraction of the grain boundary. The grain boundary plays an important role during the creep process of nanocrystalline NiAl. Indeed, the grain boundary deforms and slides more easily compared to grain interiors [26]. It is not only a good source or sink place for dislocation and vacancy, but also serves as a fast path for diffusion. Therefore, the creep rate of nanocrystalline NiAl may be increased when the volume fraction of grain boundary becomes larger in a smaller grain size model.

### 3.2. Crystal Structure Evolution on Creep Process

**Figure 3:**
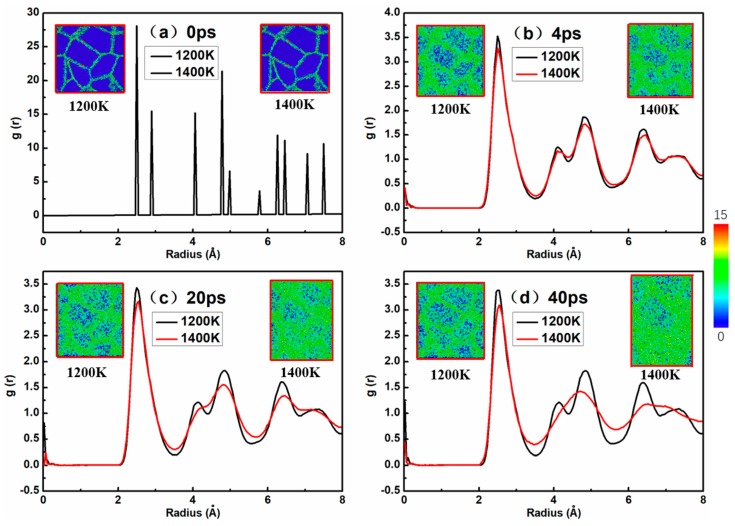
Radial distribution function plots with atomic snapshots of a representative nanocrystalline NiAl model with 4.5 nm coarser grain, colored as per CSP during creep deformation for (**a**) 0, (**b**) 4, (**c**) 20, (**d**) 40 ps under the stress levels of 2.0 GPa at 1200 and 1400 K.

As shown in Figure 3, the atomic snapshots were colored as per CSP along with the corresponding radial distribution function (RDF) of a nanocrystalline NiAl model with 4.5 nm grain size under the stress level of 2.0 GPa during the creep processes at 1200 and 1400 K. The central symmetry parameter (CSP) is used to distinguish the degree of broken inversion symmetry as compared to each atom’s local environment for a specimen under the creep deformation process. The color scale on the right expresses the degree of crystal structure damage from 0 to 15; when the value of CSP is 0, it is a perfect crystal, and the figure is represented by dark blue. The color from blue to red reflects the extent which the crystal structure was destroyed. In the atomic snapshot of Figure 3, the dark blue part represents the crystal structure while the green part represents the grain boundary structure. This is also clear from the snapshots in Figure 3. The amorphization of the model was initiated at the grain boundary atoms, where the diffusion of grain atoms was more dominant at the grain boundary than the crystal structure arranged neatly inside the model. The amorphization process then moved from the grain boundary toward the inner part of the grain as the creep deformation proceeded. From another perspective, with respect to creep temperatures, the formation of an amorphous structure occurred earlier at 1400 K compared to 1200 K. It could be seen that NiAl gradually lost its crystallinity with the progress of creep deformation, and the amorphization was more obvious after 40 ps at 1400 K, as evident from Figure 3d. The nanocrystalline NiAl model at 1200 K had a more complete crystal structure than that at 1400 K. These observations may be due to the fact that grain boundary diffusion or lattice diffusion occurs more rapidly at higher temperatures during the creep deformation process.

Notably, the first, second and third peaks can be clearly seen from the RDF curves in Figure 3. This figure refers to the coordinates of a given particle, with the probability of distribution of other particles in space; the sharp peaks represent order, and the round peaks represent disorder. The first, second, and third peaks represent the first three positions in which the particle has the highest probability of occurrence from the specified atom.

It can be seen from the curves of Figure 3 that the peak value of the RDF at 1400 K is different from that at 1200 K, especially regarding the decrease in the second and third peaks, and the peaks become wider and wider, which represents an amorphization process. This is in agreement with the visual observation in snapshots. These discrepancies are probably due to the fact that as the temperature rises, creep deformation proceeds, and thus the crystalline volume fraction is reduced and the progress of amorphization is accelerated in the model.

By investigating the relationship between the creep curve (Figure 2) and nanocrystalline NiAl model amorphization, we found that the height and shape of the RDF peaks at 1200 K did not change much with time, but the peaks at 1400 K changed significantly with time. This is because the model entered the tertiary creep stage at 40 ps and 1400 K, and the creep rate was faster than at steady-state creep. Therefore, the height of the peaks in the RDF curve dropped significantly. Another conclusion can be derived from Figure 3, namely that the crystalline structure of the model remains substantially stable when the creep deformation process is in a steady state.

### 3.3. Effects on Dislocation Evolution

**Figure 4:**
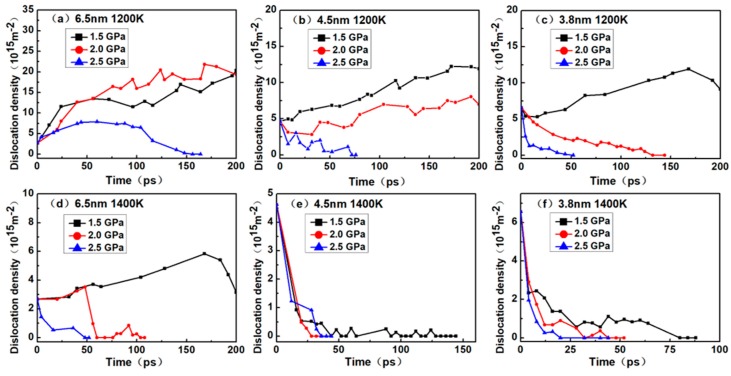
Plots of the dislocation density versus time for the nanocrystalline NiAl models with different grain sizes of (**a**) 6.5 nm at 1200 K; (**b**) 4.5 nm at 1200 K; (**c**) 3.8 nm at 1200 K; (**d**) 6.5 nm at 1400 K; (**e**) 4.5 nm at 1400 K; (**f**) 3.8 nm at 1400 K under the stress levels of 1.5, 2.0 and 2.5 GPa.

In certain creep theories, dislocation is considered to be one of the important factors influencing creep; thus, the study of dislocation density is warranted. Figure 4 shows the various dislocation densities of nanocrystalline NiAl under different simulation conditions, which explains the role of dislocations during creep process.

As shown in Figure 4a, the dislocation density gradually increased under the stress level of 1.5 GPa and 2.0 GPa. At a 2.5 GPa stress level, it increased at first and then decreased until reaching zero. In Figure 4b, the dislocation density gradually increased under the stress level of 1.5 GPa and 2.0 GPa while the dislocation densities at 2.5 GPa reduced gradually to zero. In Figure 4c, the dislocation density gradually increased under the stress level of 1.5 GPa and then decreased a little, while the dislocation densities at 2.0 and 2.5 GPa reduced gradually to zero. In Figure 4d, the dislocation densities increased gradually under the stress levels of 1.5 GPa and then reduced to the initial value. At a 2.5 GPa stress level, the value increased at first and then decreased until reaching zero, while the dislocation densities at 2.5 GPa reduced gradually to zero. In Figure 4e,f, the dislocation densities gradually reduced zero under all stress levels.

According to the creep curves in Figure 2, the dislocation density continuously increased during the creep process, indicating that the models enter primary creep and steady-state creep stages, but not the tertiary creep stage. During these stages, dislocation nucleation plays a crucial role in the deformation process of creep. When the creep curves show a tertiary creep, the dislocation density of the model may reduce to zero, and the shorter the steady-state creep stage, the faster the dislocation density decline. This can be explained by the diffusion of atoms at the grain boundaries during the tertiary creep stage, which triggers the model to lose crystallinity, hinders the dislocation movement and eventually eliminates the dislocation. The reduction and disappearance of dislocations also indicate that the creep mechanism may have been altered.

In addition, it can be seen that the initial dislocation density was independent of temperature and stress level and was solely related to the grain size. Noticeably, the smaller the nanocrystalline NiAl grain size, the greater the initial dislocation density. According to Figure 2, under the same temperature and stress conditions, the primary creep and steady-state creep stages of nanocrystalline NiAl models were shortened with decreasing grain size. Moreover, the model entering tertiary creep is considered to be advanced. Taken together, these findings suggest that the reduced grain size may contribute to the occurrence of creep during the early stage of the creep process.

### 3.4. Creep Mechanism: Diffusion and Vacancy

**Figure 5:**
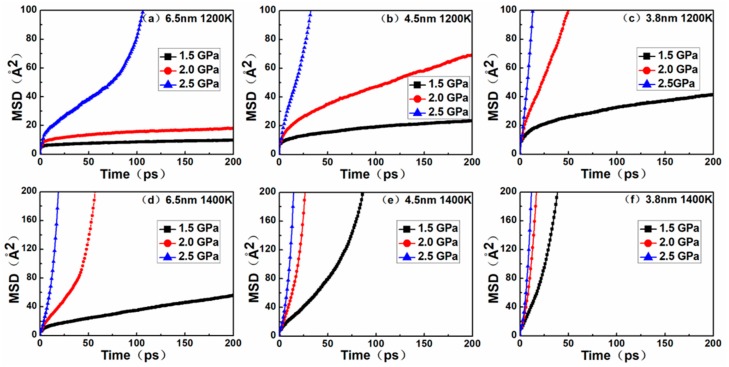
Plots of the MSD versus time for the nanocrystalline NiAl models with different grain sizes of (**a)** 6.5 nm at 1200 K; (**b**) 4.5 nm at1200 K; (**c**) 3.8 nm at 1200 K; (**d**) 6.5 nm at 1400 K; (**e**) 4.5 nm at 1400 K; (**f**) 3.8 nm at 1400 K under the stress levels of 1.5, 2.0 and 2.5 GPa.

From the above analysis, the dislocation disappeared during the creep deformation process, especially when the steady-state creep phase was completed. Figure 5 shows the MSD curves of nanocrystalline NiAl models. By comparing the MSD curves to their corresponding creep curves, it was found that the diffusion trends of atoms in the nanocrystalline NiAl were almost similar to the creep process. The results of MD simulation demonstrated that the creep deformation of nanocrystalline NiAl was significantly controlled by atomic diffusion. This is mainly due to the fact that the diffusion rate of atoms increases with decreasing grain size and increasing temperature and stress levels.

Besides this, during the creep process, the amorphous volume fraction gradually increases with time, and the amorphous structure is favorable for atomic diffusion. On the other hand, a decrease of grain size indicates an increase in the volume fraction of grain boundaries in the model, and the atoms located at the grain boundaries exhibit rapid diffusion [26]. Collectively, these findings prove that diffusion creep is one of the dominant mechanisms responsible for nanocrystalline NiAl under high temperature.

**Figure 6:**
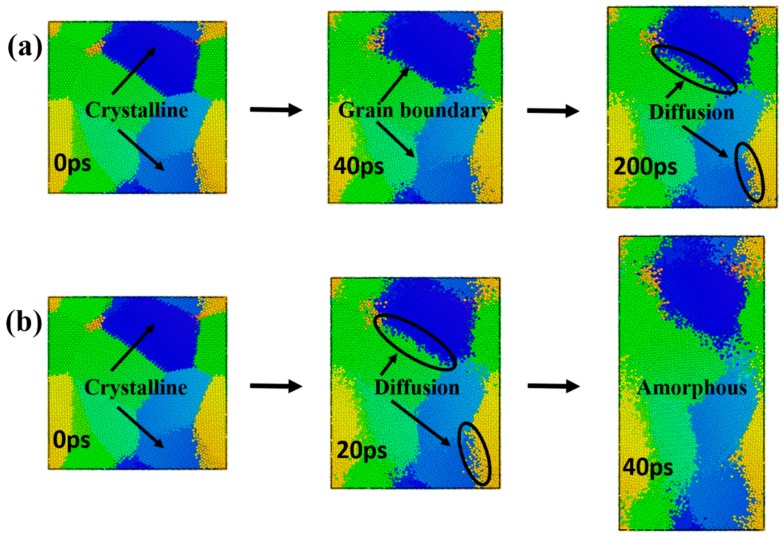
Atomic snapshots of nanocrystalline NiAl models with a grain size of 4.5 nm under the stress level of 2.0 GPa at (**a**) 1200 K and (**b**) 1400 K during the creep process at the indicated times.

In order to identify the dominant diffusion mechanism during the creep deformation process, the models with a grain size of 4.5 nm at 1200 K and 1400 K were selected under the stress levels of 2.0 GPa (see Video S1 and S2 in the Supplementary Files). Representative atomic snapshots of the microstructure changes with time are presented in in Figure 6. Color coding was applied on each grain in order to better clarify the creep mechanism. Different colors are only used to distinguish different grains, and the colors themselves are meaningless.

The creep curves corresponding to Figure 6a only demonstrated both primary creep and steady-state creep stages. As shown in Figure 6a, at 200 ps of the creep process, the atomic diffusion at the grain boundary was obvious, and the shape of the crystal grain did not change significantly. The creep curves corresponding to Figure 6b revealed a tertiary creep stage. As shown in Figure 6b, the diffusion of the grain boundary occurred first in the model. At 40 ps of this simulation, the atoms at the inner part of the grain were diffused, and the shape of the grain was elongated significantly. In accordance with the creep curve, it was found that the tertiary creep stage is accompanied by the amorphization process. It could be seen that the model exhibited grain boundary diffusion at the steady-state creep stage, and grain boundary diffusion and lattice diffusion coexisted during the tertiary creep stage (Figure 6).

**Figure 7:**
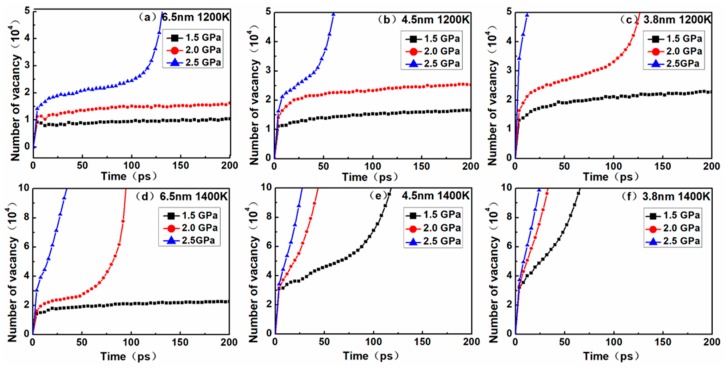
Plots of the vacancy versus time for the nanocrystalline NiAl models with different grain sizes of (**a**) 6.5 nm at 1200 K; (**b**) 4.5 nm at1200 K; (**c**) 3.8 nm at 1200 K; (**d**) 6.5 nm at 1400 K; (**e**) 4.5 nm at 1400 K; (**f**) 3.8 nm at 1400 K under the stress levels of 1.5, 2.0 and 2.5 GPa.

To deeply explore the diffusion creep mechanism, the vacancy quantity curves corresponding to the models in Figure 5 were obtained (Figure 7). Plots of the number of vacancies versus time during the creep deformation process are illustrated in Figure 7. For nanocrystalline materials, it is generally considered that the generation of vacancy is one of the main causes underlying the diffusion creep mechanism, owing to the larger volume fraction of grain boundaries in nanocrystalline materials and the higher probability of vacancies at grain boundaries. In this study, point defects such as vacancy were identified using Wigner–Seitz cell method by comparing the defective crystal with a corresponding perfect crystal lattice and distinguishing a vacancy site from non-atom occupying sites.

Through the comparison between Figure 5 and Figure 7, it was found that although the vacancy quantity curve and the MSD curve exhibited a similar trend, the time nodes of the curve turning points were somewhat different. Possible explanations for this discrepancy are as follows. In the nanocrystalline material, as creep deformation progresses, the volume fraction of the amorphous structure increasingly accumulates at the grain boundary, and thus a massive number of vacancies are generated. In general, it is relatively easier for atoms to diffuse through vacancies than by substitution, but at sufficiently high temperatures and stress levels, atoms have enough energy to break through barriers and carry out lattice diffusion in the form of substitution. Therefore, the MSD curves not only contain the results of atomic diffusion through vacancies, but also the diffusion of atoms via substitution. These two creep mechanisms can be evidenced from the atomic snapshots of Ovito in Figure 6.

In order to more accurately determine the creep mechanism in the creep process, it is necessary to discuss the relationship between dislocation and vacancy. By comparing the curves between Figure 4 and Figure 7, it was found that the dislocation density increased slowly, and the number of vacancies also increased slowly. This phase corresponded to the primary and steady-state phases of the creep process, indicating that there was a dislocation mechanism in this creep phase. When the dislocation density decreased to zero, it could be seen that the corresponding number of vacancies increased rapidly. This phase corresponded to the tertiary creep phase of the creep process, indicating that the dominant mechanism of the creep process was the diffusion mechanism rather than the dislocation mechanism.

It is worth noting that our findings are consistent with the plausible creep mechanisms. These findings also support the existence of two diffusion mechanisms of grain boundary diffusion and lattice diffusion in nanocrystalline NiAl. Based on the above analysis, it can be found that the transformation mechanisms of nanocrystalline NiAl in different creep stages are in agreement with the findings of Meraj and Pal [12,24], indicating that the transformation mechanisms tend to exhibit a universality for the creep of nanocrystalline metal at high temperature.

## 4. Conclusions

In this study, the effects of temperature, stress and grain size on the creep process of nanocrystalline NiAl were investigated through MD simulation. Specifically, the creep mechanisms corresponding to different creep stages were revealed, and the following conclusions were drawn:

(1) From creep curves, it could be found that with increasing temperatures and stress levels and decreasing grain sizes, the creep rate was higher and the tertiary creep occurred earlier.
(2) The regular changes in dislocations, vacancies and grain boundaries were observed at different stages of nanocrystalline NiAl creep curves, indicating that dislocation, vacancy and the grain boundary play a pivotal role during the creep deformation process of nanocrystalline NiAl.
(3) Through the RDF curves of nanocrystalline NiAl and the atomic snapshots, it was observed that the higher the temperature, the earlier the occurrence of amorphization, and the amorphization was more obvious in the tertiary creep stage compared to primary creep and steady-state creep stages. During the steady-state creep phase, the crystal structure of the model remained stable. The model gradually became an amorphous solid, indicating that the creep process occurred at the tertiary stage.
(4) During primary creep and steady-state creep stages, atomic diffusion at the grain boundary could be seen, and the dislocation density increased gradually, indicating that the creep mechanism at these stage was Coble creep controlled by grain boundary diffusion while accompanied by dislocation nucleation. In tertiary creep, the dislocation density decreased exponentially, along with the amorphization of the model. It was noted that some atoms diffused across the vacancies, and the remaining atoms diffused through subrogation, indicating that the creep mechanisms at this stage were Coble creep controlled by grain boundary diffusion and Nabarro–Herring creep controlled by lattice diffusion.

## Supplementary Materials

The following are available online at https://www.mdpi.com/1996-1944/12/16/2508/s1, For Video S1, it is the nanocrystalline NiAl model with a grain size of 4.5 nm at 1200 K under the stress levels of 2.0 GPa. As can be seen from the creep curve on the right, the tertiary creep stage did not occur during the 200 ps. In the primary creep and steady-state creep stages processes, it can be seen from the left part that the shape of the grains did not change significantly, and atomic diffusion occured only at the grain boundaries, indicating that in this simulation only happened grain boundary diffusion. For Video S2, it is the nanocrystalline NiAl model with a grain size of 4.5 nm at 1400 K under the stress levels of 2.0 GPa. It can be seen from the creep curve on the right, the model entered the tertiary creep stage after 20ps. As can be seen from the left part, the shape of the grains in the model were obviously elongated after 20ps, and the diffusion phenomenon was gradually from the grain boundary extend to the inside of the grains, indicating that grain boundary diffusion and lattice diffusion were existed simultaneously in the tertiary creep stage.
